# Prevalence and Phylogeny of Coronaviruses in Wild Birds from the Bering Strait Area (Beringia)

**DOI:** 10.1371/journal.pone.0013640

**Published:** 2010-10-29

**Authors:** Shaman Muradrasoli, Ádám Bálint, John Wahlgren, Jonas Waldenström, Sándor Belák, Jonas Blomberg, Björn Olsen

**Affiliations:** 1 Section of Clinical Virology, Department of Medical Sciences, Uppsala University, Uppsala, Sweden; 2 Section for Bacteriology and Food Safety, Department of Biomedical Sciences and Veterinary Public Health, Swedish University of Agricultural Sciences, Uppsala, Sweden; 3 The Joint Research and Development Division, Departments of Virology and Parasitology, Swedish University of Agricultural Sciences and the National Veterinary Institute, Uppsala, Sweden; 4 Department of Microbiology, Tumor and Cell Biology, Karolinska Institutet, Stockholm, Sweden; 5 Swedish Institute for Infectious Disease Control, Solna, Sweden; 6 Section for Zoonotic Ecology and Epidemiology, Linneaus University, Kalmar, Sweden; 7 Section of Infectious Diseases, Department of Medical Sciences, Uppsala University, Uppsala, Sweden; Nanyang Technological University, Singapore

## Abstract

Coronaviruses (CoVs) can cause mild to severe disease in humans and animals, their host range and environmental spread seem to have been largely underestimated, and they are currently being investigated for their potential medical relevance. Infectious bronchitis virus (IBV) belongs to gamma-coronaviruses and causes a costly respiratory viral disease in chickens. The role of wild birds in the epidemiology of IBV is poorly understood. In the present study, we examined 1,002 cloacal and faecal samples collected from 26 wild bird species in the Beringia area for the presence of CoVs, and then we performed statistical and phylogenetic analyses. We detected diverse CoVs by RT-PCR in wild birds in the Beringia area. Sequence analysis showed that the detected viruses are gamma-coronaviruses related to IBV. These findings suggest that wild birds are able to carry gamma-coronaviruses asymptomatically. We concluded that CoVs are widespread among wild birds in Beringia, and their geographic spread and frequency is higher than previously realised. Thus, Avian CoV can be efficiently disseminated over large distances and could be a genetic reservoir for future emerging pathogenic CoVs. Considering the great animal health and economic impact of IBV as well as the recent emergence of novel coronaviruses such as SARS-coronavirus, it is important to investigate the role of wildlife reservoirs in CoV infection biology and epidemiology.

## Introduction

Coronaviruses (CoVs), members of the Coronaviridae family and sub family Coronavirinae within the order Nidovirales, are enveloped viruses with a positive-sense RNA genome of 27–31 kb [1]. Based on genetic and serological analyses, CoVs are divided into three genera [2] alpha- and beta- and gamma-coronaviruses.

Alpha- and beta-coronaviruses have been isolated from mammals, while gamma-coronaviruses genera is formed by Avian infectious bronchitis virus (IBV), together with the genetically closely related Turkey coronavirus [3], [4], [5], [6], Pheasant coronavirus [7], and recently identified coronaviruses from several species of wild birds [8], [9], a beluga whale [10] and an Asian Leopard cat [11].

Due to their high mutation and recombination rate during replication, CoVs are able to generate extensive genotypic variation, which facilitates adaptation to new host species [12]. In animals, CoVs generally cause respiratory or intestinal infections, but they have also been associated with a wide spectrum of other clinical symptoms, including hepatic, renal, reproductive and neurological diseases [13]. The only exception is the notorious feline infectious peritonitis virus (FIPV), which causes a sporadic but fatal generalized disease in Felidae [14].

Beyond their causal role in the common cold, human coronaviruses (HCoVs) received relatively little attention as human pathogens until the emergence of the severe acute respiratory syndrome (SARS) epidemic in 2003 [15]. The identification of SARS-CoV, with its pandemic potential, provided new impetus to CoV research; since then, many previously unidentified CoVs have been discovered in humans and animals [16], [17]. Because evidence based on molecular genetic data showed that the causative agent of SARS most likely originated from recombination events between mammalian-like and avian-like parental viruses present in wild animal species [18], CoVs are considered not only as pathogens of veterinary importance but also as a threat to mankind. Therefore, surveillance for possible animal reservoirs of CoVs has gained importance in the research community.

Wild bird species are reservoirs for a number of emerging viruses. The most well-known among them is avian influenza A virus [19]. Wild bird species may also harbour other respiratory and enteric viruses, including CoVs. IBV is a gamma-coronavirus that is responsible for severe economic losses in the poultry industry [20]. This global virus causes an acute and highly contagious respiratory disease in chickens (*Gallus gallus*) of all ages and diminishes egg production in hens [21]. A number of IBV strains cause severe nephritis, with mortality reaching up to 30% [20]. Disease control is based mostly on vaccination, but the virus constant antigenic changes sometimes result in incomplete protection [22]. Although chickens are the primary natural host of IBV, several research groups have recently found IBV-like viruses and new CoVs in other bird species [13], [23]. Furthermore, new CoVs that are genetically distinct from IBV have been identified in different bird families [9] and in beluga whales [10]. Our investigations showed the presence of gamma-CoVs in wild mallards (*Anas platyrhynchos*), indicating that wild ducks may spread and harbour CoVs [24]. These data emphasize that the geographic distribution, host range and genetic diversity of avian CoVs are much greater than was previously thought, a finding that could impact both animal and human health.

In the present study, we performed surveillance and molecular epidemiological studies on CoV infections in wild birds from the Beringia area (between Siberia and Alaska) for the following reasons: (1) to study the genetic diversity of avian viruses with a special emphasis on new CoV subgroups; (2) to characterise the distribution of CoVs in an area where the Arctic meets the Pacific to better understand how the viruses may emerge and spread globally; (3) to study the phylogenetic aspects of CoVs in wildlife reservoirs.

## Results

### Prevalence of coronaviral nucleic acid in wild birds

Samples originating from 26 bird species were tested for CoVs polymerase (RdRp) gene by RT-PCR, and 64 of the 1,002 faecal and cloacal samples were positive (6.4%). Positives were found in 18 species. We classified the species into six different groups that reflected both their taxonomy and their ecology. These groups were geese (5 species, n = 233), waders (9 species, n = 130), gulls (6 species, n = 322), ducks (1 species, n = 122), auks (2 species, n = 128) and seabirds (3 species, n = 67) (**Table 1**). Gamma-coronaviruses were found in all the 18 CoV positive bird species, and there was a significant difference in PCR-prevalence between bird groups (χ^2^ = 36, 43, df = 5, p<0.001). Wader species were most frequently noted as CoV-positive (17.1%), followed by ducks (11.5%), geese (8.2%), gulls (3.1%) and seabirds (1.5%), while auks had the lowest prevalence (0.8%). In contrast, none of the 101 examined tufted puffins were CoV positive. Furthermore, there was notable variation in the proportion of PCR-positive samples between areas: Point Barrow, Kolyushin Commander- and Wrangel Islands, Petropavlovsk-Kamchatskiy. The lowest rates were observed on Commander (2.3%) and Wrangel Islands (3.5%); Petropavlovsk-Kamchatskiy was at the mid-point (8.2%), and the highest rates were detected at Point Barrow (11%) and Kolyushin (27.5%) (χ^2^ = 81.51, df = 4, p<0.001) (**Table 2**). However, the sampled avifauna also differed among sites (χ^2^ = 1754.7, df = 20, p<0.001); while ducks were only sampled at Point Barrow, the majority of waders and geese were sampled from the tundra at Kolyushin and Wrangel Island (**Table 3**).

**Table 1:** Overview of wild bird samples involved in the study, and the prevalence of CoV in different species.

Order	Group	Species	Positive (n)	Sampled (n)	Rate %
**Anseriformes**	**Geese**	**5 species**			
		*Anser canagica*	10	22	45.5
		*Branta bernicla*	1	11	9.1
		*Anser caerulescens*	7	188	3.7
		*Anser albifrons*	1	11	9.1
		*Anser anser*	-	1	0
	**Ducks**	**1 Species**			
		*Anas acuta*	14	122	11.5
**Charadriiformes**	**Waders**	**9 Species**			
		*Calidris melanotos*	-	1	0
		*Phalaropus fulicarius*	3	14	21.4
		*Calidris or Erolia ruficollis*	7	75	9.3
		*Eurynorhynchus pygmeus*	1	1	100
		*Calidris mauri*	5	23	21.7
		*Calidris alpina*	1	5	20.0
		*Calidris pusilla*	1	5	20.0
		*Phalaropus lobatus*	1	5	20.0
		*Arenaria interpres*	-	1	0
	**Gulls**	**6 Species**			
		*Larus ridibundus*	5	61	8.2
		*Larus glaucescens*	2	148	1.4
		*Larus vegae*	2	36	5.6
		*Larus hyperboreus*	1	11	9.1
		*Rissa brevirostris*	-	65	0
		*Rissa tridactyla*	-	1	0
	**Auks**	**2 Species**			
		*Fratercula cirrhata*	-	101	0
		*Cepphus columba*	1	27	3.7
**Pelacaniformes**	**Seabirds**	**3 Species**			
		*Phalacrocorax sp*	1	48	2.1
		*Phalacrocorax urile*	-	18	0
		*Puffinus tenuirostris*	-	1	0
**Total: 3**		**26**	**64**	**1002**	**6.4**

**Table 2:** Prevalence of avian CoV in five geographic sites in the Beringia area.

Sampling site	Positive (n)	Sampled (n)	Rate (%)
**Point Barrow**	18	163	11
**Kolyushin**	22	80	27.5
**Wrangel Island**	8	226	3.5
**Commander Island**	11	472	2.3
**Petropavlovsk-Kamchatskiy**	5	61	8.2
**Total**	**64**	**1002**	**6.4**

**Table 3:** Proportion of sampled avifauna across the sites.

Group	Kolyushin	Point Barrow	Wrangel Island	Commander Island	Petropavlovsk-Kamchatskiy	Total
**Geese**	22	12	199	-	-	233
**Waders**	48	18	1	63	-	130
**Gulls**	10	11	26	214	61	322
**Ducks**	-	122	-	-	-	122
**Auks**	-	-	-	128	-	128
**Seabirds**	-	-	-	67	-	67
**Total**	**80**	**163**	**226**	**472**	**61**	**1002**

### Phylogenetic analyses

**Figure 1:**
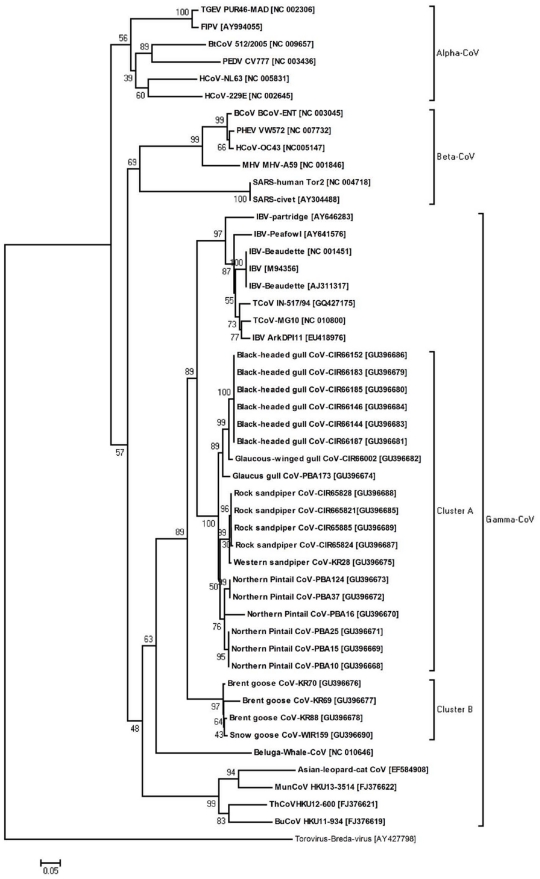
Neighbour-joining tree of CoVs based on a 560-nt fragment (excluding primer sequences) of the CoV RNA-dependent RNA polymerase. The significance of the tree topology was assessed by 1,000 bootstrapping steps. Evolutionary distances were computed using the Kimura 2 parameter model and are given in the number of base substitutions per site. Previously published mammalian and avian CoV sequences are also included for comparison. *BuCoV* bulbul coronavirus; *BCoV* bovine coronavirus; *FIPV* feline infectious peritonitis virus; *IBV* infectious bronchitis virus; *HCoV* human coronavirus; *MunCoV* munia coronavirus; *MHV* murine hepatitis virus; ThCoV thrush coronavirus; *TGEV* transmissible gastroenteritis virus; *SARS* severe acute respiratory syndrome; *PEDV* porcine epidemic diarrhea virus; *PHEV* porcine hemagglutinating encephalomyelitis virus; *FIPV* feline infectious peritonitis virus. The sample identification includes; bird species, virus, site, location and sample identification number. *PBA* Point barrow Alaska; *KR* Kolyuchin Russia; *CIR* Commander Island Russia; *WIR* Wrangel Island Russia. Within the gamma-coronaviruses branch, two clusters were identified. Cluster A contained eight Alaskan and one Russian virus, and the within-group distance ranged between 0.8-16%. The Russian Brent goose viruses formed cluster B with a within-group distance of 0.9-3.5%. GenBank accession numbers are indicated in brackets.

Phylogenetic analyses of amplified CoV polymerase (RdRp) gene fragments showed that the detected viruses belonged to gamma-coronaviruses. Trees constructed using the neighbour-joining, maximum parsimony and maximum likelihood methods all showed similar topologies (**Figure 1** and data not shown). All of the viruses found in this study belonged to gamma-coronaviruses and were most closely related to IBV strains (nucleotide distances ranged between 17.9–24.2%). Within the gamma-coronaviruses branch, two clusters were identified. Cluster A contained eight Alaskan and one Russian virus, and the within-group distance ranged between 0.8–16%. The Russian Brent goose viruses formed cluster B with a within-group distance of 0.9–3.5%. The mean distance between the two clusters was 19.1%. The trees also revealed clustering of strains based on geographic location and host species. The Alaskan and Russian CoVs formed distinct clusters in the majority of the trees. However, the trees also revealed diversity among CoV strains originating from the same geographic area. The goose and duck viruses clustered separately, but CoVs of other origins showed more extended genetic diversity.

## Discussion

In the present study, gamma-coronaviruses were detected in 6.4% of the examined wild bird samples, with some variation between geographically separated populations. An example of a major difference between hosts within CoV phylogenies is the gamma-coronaviruses, which contains both avian CoVs and CoVs isolated from beluga and Asian leopard cats. Beluga lives in the arctic waters around the sampling sites of this investigation. This might indicate a degree of bird-mammalian CoV exchange. Taken together, the data show that there is circulation of genetically divergent avian CoVs among the wild bird population in the Beringia region.

Although the number of samples analyzed in this study was limited, it can be assumed that the genetic variation of CoVs among wild birds is much higher than previously thought. The detection of coronaviruses related to the gamma-coronaviruses in geographically distinct areas, such as Russia and Alaska, indicate that CoVs are widespread among birds associated with water environments, and this may have implications for poultry health. Our results show that gamma-coronaviruses are much more widespread among birds than was previously suspected. Most of the birds sampled in this study are migratory and leave Beringia when conditions deteriorate in the autumn. Some birds, such as the gulls and auks, remain at fairly northern latitudes, while the ducks, geese and waders migrate either to Asia, North America or even South America. The Beringia area has been proposed to be an important gateway between Eurasia and North America for influenza A virus, where the meeting of birds and viruses from different hemispheres can allow disease transmission to occur [25]. From this and other studies, it is reasonable to assume that a great variety of hitherto undetected CoVs exist in wild bird species. Previous studies that examined the host range and genetic diversity of CoVs [23], [26], [27] have revealed that CoVs in wild birds are present mainly in wildfowl (*Anseriformes*) and waders (*Charadriiformes*). Our results corroborate these findings and indicate that CoVs are common among birds in the Beringia region. Intensified surveillance of wild birds is an important means of assessing the relative prevalence of IBV strain variants, and this knowledge would aid risk assessments and risk management of these viruses. Wild bird surveillance that includes virus isolation may also be a tool for obtaining strains of avian CoV that can be used for vaccine development and diagnostics, as some of these sequences are indeed similar to outbreak strains of IBV.

The CoVs detected in this study were genetically diverse within the examined genomic region. All the viruses were phylogenetically close to various IBV strains, and all belonged to gamma-coronavirusa. An important question is whether these viruses originated from IBV [26], [27] of domesticated birds, or whether wild birds were the original reservoirs and IBV emerged from the wildlife. Some support for the latter notion comes from the three gamma-coronavirusa Russian Brent goose CoVs that showed a considerable phylogenetic distance from IBV and were more closely related to goose coronavirus [8]. However, more genetic data are needed to conclusively resolve this question. Since avian coronaviruses get their diversity primarily from hypervariable regions residing in the spike gene, highly detailed genetic characterization of this genomic region including metagenomic methods will provide information regarding the phylogenetic relationship between different gamma-coronaviruses.

Taken together, this study provides insight into the genetic diversity of avian CoVs including its wildlife animal reservoirs. The majority of the human emerging infectious diseases of the past few decades, including AIDS, Ebola fever, avian influenza and severe acute respiratory syndrome (SARS), resulted from interspecies transmission of zoonotic RNA viruses [28], [29], [30], [31]. Adaptation of a non-human CoV to a human host occurred with both SARS-CoV and OC43-CoV; both are examples of a penetration of the animal-human species barrier. It is likely that they were enzootic in an unknown animal species before suddenly emerging as a virulent human virus. Before establishing the ecology of the emergence of these human viral pathogens and reconstructing their evolutionary pathways, it is necessary to identify closely related CoVs in wild animal hosts [32]. Examining the prevalence and effects of CoV infections in wild birds will increase our knowledge about CoV interactions with their hosts and may suggest as yet unexploited avenues for combating CoV infections. There is a clear need for a better understanding of CoV ecology, and this will require more data through better surveillance of wild birds and more research on the behaviour of these viruses in wild bird populations.

## Materials and Methods

### Ethical Statement

All animals were handled in strict accordance with good animal practice as defined by the Laboratory Animal of Swedish Board of Agriculture, and all animal work was approved by the Russian Academy of Medical Sciences and Russian Authorities.

### Sampling

**Figure 2:**
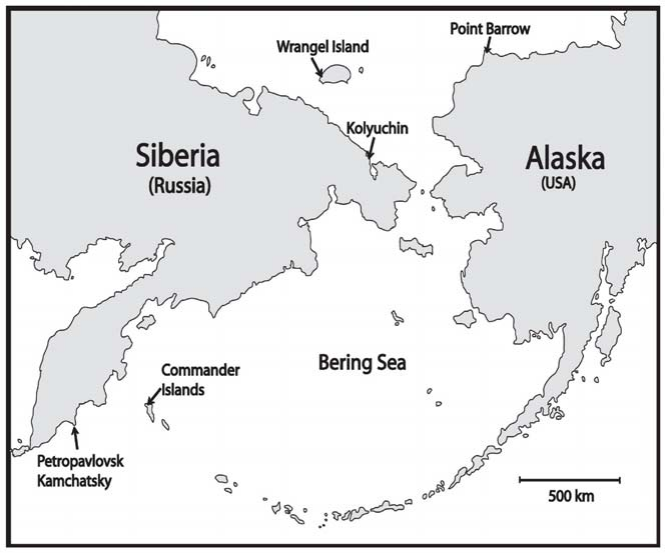
Map of Beringia showing locations where wild bird samples analysed in this study were collected.

In total, 1,002 samples were collected from five geographic areas within the Bering Strait: Point Barrow, Kolyushin, Wrangel Island, Commander Island and Petropavlovsk-Kamchatskiy (**Figure 2**). These samples represented 26 different bird species, three bird orders and six bird families (**Table 4**). The samples were collected either with a sterile cotton swab from the cloacae of trapped birds (shorebirds) or from fresh faecal samples on the ground (northern pintails *Anas acuta*). Shorebirds were caught using foldable walk-in traps [33], and northern pintails were sampled by collecting droppings after flushing the birds from sandbanks. For faecal sample collection, we made sure that the flock to be sampled contained only northern pintails by watching them with a telescope, and only fresh droppings were sampled. Each sample was immediately stored in Hank's balanced salt solution (HBSS) [34] and maintained at −80°C until the samples reached the laboratory.

**Table 4:** Overview of wild bird samples involved in the study.

Order	Family	Species (n)	Sampled (n)
**Anseriformes**	Anatidae	6	355
**Charadriiformes**	Scolopacidae	9	339
	Laridea	6	113
	Alcidae	2	128
**Pelacaniformes**	Phalacrocoracidae	2	66
	Procellariidae	1	1
**Total:3**	**6**	**26**	**1002**

### Detection of coronavirus by RT-PCR

Virions were lysed by mixing a 150-µl sample with 450 µl TRIzol (Invitrogen, Carlsbad, CA, USA). Nucleic acid was subsequently separated by the addition of 160 µl chloroform to yield an excess of 300 µl water phase suitable for RNA purification. Viral RNA was extracted using the MagAttract Viral RNA M48 extraction kit in combination with the M48 Biorobot (both supplied by Qiagen, Hilden, Germany). The CoV screening was performed by amplifying a 179-nt stretch of the CoV RdRp gene using our previously published one-step pan-CoV RT-PCR method [24]. Standard precautions were taken to avoid PCR contamination, and no false-positive result was observed in water controls.

### cDNA synthesis and sequencing

cDNA was synthesized from RNA from all of the pan-CoV RT-PCR positive samples in 50-µl reactions with l0 µl of RNA. cDNA synthesis was performed on a Cycler IQ™ PCR-cycler (Bio-Rad Laboratories, Hercules, CA, USA) at 25°C for 10 min, 37°C for 90 min and 70°C for 15 min. The 50-µl reactions contained 1× AffinityScript buffer (Stratagene, La Jolla, CA, USA), 10 mM DTT (Promega, Madison, WI), 800 µM dNTPs (Applied Biosystems, Foster, CA, USA), 530 ng random hexamers (GE Healthcare, Piscataway, NJ, USA) and 50 U AffinityScript reverse transcriptase (Stratagene). The cDNA samples were used to amplify a 608-610-bp stretch in the polymerase gene using degenerate primers: forward primer (5′-TGGGWTGGGAYTAYCCWAARTGYGA-3′) and reverse primer (5′-GCATWGTRTGYTGNGARCARAATTC-3′). The forward primer was used as previously published by Woo et al. [9] with slight modifications. The reverse primer was published by Escutenaire et al. and Muradrasoli et al.[24], [35]. The PCR products were gel-purified using a QIAquick gel extraction kit (QIAgen, Hilden, Germany). Both strands of the PCR products were sequenced using the fluorescent dye terminator method with an ABI PRISM® Big Dye™ Terminator Cycle Sequencing v3.1 Ready Reaction kit (Perkin Elmer, Waltham, MA, USA) on an ABI PRISM® 310 genetic analyzer according to the manufacturer's recommendations (Applied Biosystems).

### Statistical analysis

We used chi-square tests to determine the between-site effects and the effect of bird group on the proportion of positive individuals. A value of P<0.001 was considered statistically significant.

### Sequence and phylogenetic analysis

Sequences were assembled and edited using the BioEdit v.7.0.7. [36] and the DNASTAR 7 (Lasergene, WI, USA) software packages. Identification was performed using the Basic Local Alignment Search Tool (BLAST) to search for sequences available at the National Center for Biotechnology Information in Bethesda, Maryland, USA (http://www.ncbi.nlm.nih.gov). Distance based neighbour-joining and character based maximum parsimony phylogenetic trees were generated using the Molecular Evolutionary Genetics Analysis (MEGA) software v.4.0. [37]. The neighbour-joining algorithm was implemented with the Kimura-2 parameter model using a transition-to-translation ratio of 2.0. Other models were tested that showed similar topologies. The topology of the trees was confirmed by 1,000 bootstrap replicates. Maximum likelihood phylogenetic trees were generated using the PHYLIP version 3.68 software [38]. The sequences were deposited in GenBank under the following accession numbers: GU396668-GU396690.
